# Loss of Gene Information: Discrepancies between RNA Sequencing, cDNA Microarray, and qRT-PCR

**DOI:** 10.3390/ijms22179349

**Published:** 2021-08-28

**Authors:** Nicole Rachinger, Stefan Fischer, Ines Böhme, Lisa Linck-Paulus, Silke Kuphal, Melanie Kappelmann-Fenzl, Anja K. Bosserhoff

**Affiliations:** 1 Institute of Biochemistry, Friedrich-Alexander University Erlangen-Nürnberg (FAU), Fahrstraße 17, 91054 Erlangen, Germany; nicole.rachinger@fau.de (N.R.); ines.boehme@fau.de (I.B.); lisa.linck@fau.de (L.L.-P.); silke.kuphal@fau.de (S.K.); 2 Faculty of Computer Science, Deggendorf Institute of Technology, Dieter-Görlitz-Platz 1, 94469 Deggendorf, Germany; stefan.fischer@th-deg.de (S.F.); melanie.kappelmann-fenzl@fau.de (M.K.-F.)

**Keywords:** transcriptome analysis, RNA sequencing, cDNA microarray, qRT-PCR, secondary structure, free energy of the RNA secondary structure

## Abstract

Molecular analyses of normal and diseased cells give insight into changes in gene expression and help in understanding the background of pathophysiological processes. Years after cDNA microarrays were established in research, RNA sequencing (RNA-seq) became a key method of quantitatively measuring the transcriptome. In this study, we compared the detection of genes by each of the transcriptome analysis methods: cDNA array, quantitative RT-PCR, and RNA-seq. As expected, we found differences in the gene expression profiles of the aforementioned techniques. Here, we present selected genes that exemplarily demonstrate the observed differences and calculations to reveal that a strong RNA secondary structure, as well as sample preparation, can affect RNA-seq. In summary, this study addresses an important issue with a strong impact on gene expression analysis in general. Therefore, we suggest that these findings need to be considered when dealing with data from transcriptome analyses.

## 1. Introduction

To examine the expression of individual genes, quantitative RT-PCR (qRT-PCR) is a widely used method. However, in the past, it became more and more important to analyze the complete transcriptome within cells in an unselected manner for an in-depth understanding of, e.g., development and disease [1]. Starting in 1995, microarrays were used as a standard method for analyzing parts of the transcriptome, until 2008, when the next-generation sequencing method, RNA-sequencing (RNA-seq), revolutionized research [2].

DNA microarray analyses, which are based on the hybridization of fluorescently labeled probes, require a reference genome/transcriptome for designing the microarray probes. Therefore, cDNA microarrays are dependent on the choice of probes and their binding specificity. Here, background signals and cross-hybridization can reduce the specificity and sensitivity for some genes, which can lead to pitfalls and misallocations during an analysis [3,4,5,6,7].

Due to these limitations of microarrays and the advantages of RNA-seq, such as cost reduction through massively parallel sequencing, a shift from cDNA array analysis to RNA-seq experiments has been observed in recent years [1,2,8,9]. Nowadays, RNA-seq is a frequently used method with increasing importance for studying differential gene expression, analyzing alternative splicing, and identifying novel RNA species [7,10,11]. Moreover, this technique enables a wider dynamic range for detection and provides less background noise [7]. Despite all of its advantages, RNA-seq is still a technology that is in development, and its challenging aspects range from sample preparation (fragmentation method, size selection, library construction) to computational analysis (alignment, quantification, filtering, and normalization), which have a great impact on the overall final results [10,12].

Generally, transcriptome analyses, especially of large sample sets, are usually performed with the newest and most advanced methods. Hardly ever is the same sample set analyzed with both methods in parallel. So far, only a few comparisons that indicate possible discrepancies between the different methods for transcriptome analyses exist [13,14].

In this study, we compared the detection of two groups of genes (the *SOX* gene family and *YAP/TAZ* effectors of the Hippo pathway) in various melanoma cell lines with different transcriptome analysis methods—cDNA array, RNA-seq, and classical quantitative RT-PCR—and revealed striking differences in gene detection. When comparing the expression analysis results using these techniques, we observed that some genes were detectable with qRT-PCR and cDNA arrays, but not with RNA-seq, and vice versa. In this study, we focused on analyzing the loss of information within RNA-seq and, therefore, compared our transcriptome data in different melanoma cell lines with the qRT-PCR and cDNA microarray data, as well as with datasets from different research groups. Our results demonstrate the urgent need for an in-depth understanding regarding RNA-seq data and the related methodological challenges, and we show that there are variances in outcomes with different processing methods.

We herewith suggest that the results of one methodology, which may be state of the art, should always be validated with a second methodology with regard to the experimental setting.

## 2. Results and Discussion

### 2.1. Correlation of RNA-Seq and cDNA Microarrays

In recent years, several groups, such as Fu et al. or Zhao et al., to name a few, developed algorithms for comparing transcriptome data from microarray analyses and RNA-seq [15,16]. In this study, however, we do not intend to present an algorithm for the direct comparison of microarray fluorescence signals with read counts from RNA-seq, but rather to point out, in general, that the two methods can reveal discrepancies for the detection of individual genes.

**Figure 1:**
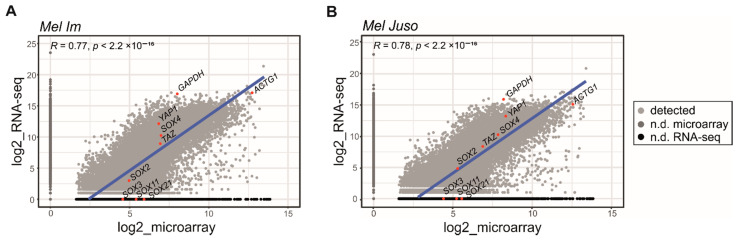
Scatter plots depicting the detected genes measured with two different methods for transcriptome analysis, shown for the melanoma cell line *Mel Im* in (**A**) and *Mel Juso* in (**B**). The measured signals with log2 are depicted as plots for each gene. The light gray color shows genes that were measured with both methods, the dark gray color shows genes that were not detected (n.d.) with microarray analysis, and the black dots depict genes that were not detected with RNA-seq (genes that were not detected by RNA-seq are listed in Supplementary Table S3). The blue line represents the Spearman correlation between genes that were detected with both methods. The genes named within the plots (labeled in red) are further discussed within the following text. Statistical analysis was performed using R.

To define the main variations between the results of a cDNA microarray (cDNA array) and RNA-seq, we first analyzed the differences in gene expression obtained by these two methods exemplarily based on melanoma cell lines. We compared the transcriptome analysis results (Supplementary Tables S1 and S2) and performed correlation analyses, which are illustrated as scatter plots (Figure 1). We determined that the gene expression in the melanoma cell line *Mel Im* was only detectable with RNA-seq, which was expected due to its methodological advantages with respect to microarray analysis (Figure 1A). Surprisingly, we also found genes that were only detectable with microarray analysis, but not with RNA-seq (Figure 1A, Supplementary Table S3). To demonstrate that this observation is not specific to the cell line, we compared the transcriptome data for another melanoma cell line, *Mel Juso* (Figure 1B, Supplementary Table S3). Here, there were also genes that were detectable with one method and not with the other transcriptome analyses. In previous studies focusing on the analysis of gene expression with different methods, the loss of gene detection with RNA-seq was not described [13,14]. Due to the unexpected result that genes were not found within the RNA-seq data, but were detectable with the cDNA array analysis, we confirmed our findings by comparing the gene expression observed with microarray or RNA-seq by focusing on individual genes (Figure 1, red label). (The reverse analysis of genes detected with RNA-seq that were not found with the microarray was neglected because of the specific choice of genes analyzed in the microarray.)

### 2.2. Analysis of the Loss of SOX Genes with RNA-Seq

**Figure 2:**
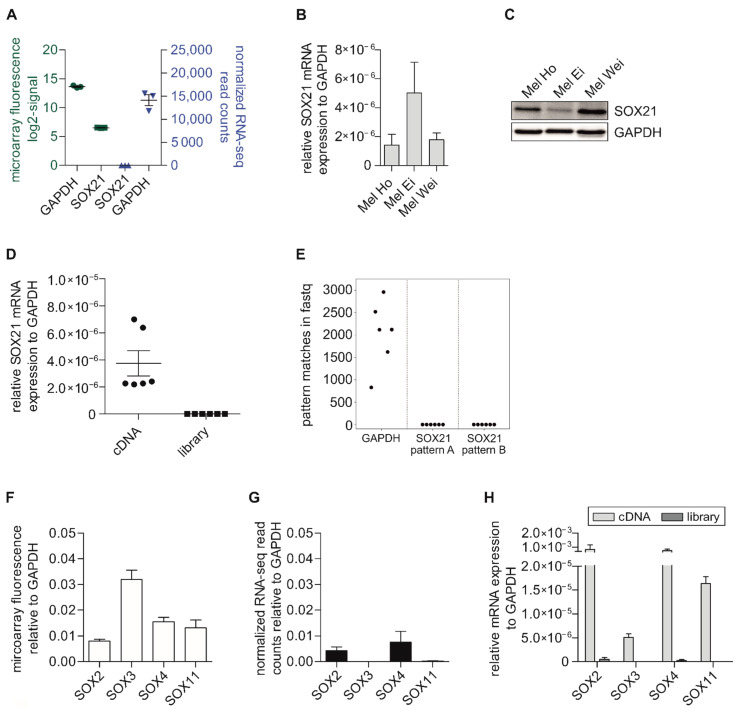
Comparison of cDNA microarray analysis with RNA-seq and qRT-PCR measurements for different *SOX* genes. (**A**) Evaluation of the raw data from transcriptome analysis with a cDNA microarray (green, Supplementary Table S4) and RNA-seq (blue, Supplementary Table S5) for *GAPDH* and *SOX21*. (**B**) Expression analysis of *SOX21* mRNA with qRT-PCR in different melanoma cell lines. (**C**) Western blot analysis revealing the SOX21 protein levels in human melanoma cell lines. (**D**) Exemplary analysis of *SOX21* mRNA expression in cDNA and library samples with qRT-PCR for two melanoma cell lines (*Mel Juso*, *Mel Wei*). (**E**) The absolute numbers of pattern matches with no mismatches are shown for the *GAPDH* and two *SOX21* patterns for the *Mel Im*, *Mel Wei*, and *Mel Juso* cell lines. Both paired-end fastq files per cell line were used for the pattern search. (**F**) Evaluation of the microarray fluorescence signal for further *SOX* genes relative to *GAPDH* based on cDNA array data (Supplementary Table S4). (**G**) Representation of the normalized RNA-seq read counts for further *SOX* genes relative to *GAPDH* (Supplementary Table S5). (**H**) Comparison of qRT-PCR measurements for further *SOX* genes of cDNA and library samples analogous to Figure 2D. For all analyses (**A**–**H**), at least two different melanoma cell lines were used with three biological replicates. *GAPDH* was used as an internal control for each transcriptome method and cell line separately. The dot and box plots show the mean ± SEM.

Next, we aimed to identify the possible causes of the observed loss of some of the aforementioned genes (Figure 1A,B, labeled in red). We first analyzed the expression of the housekeepers (*glyceraldehyde-3-phosphate dehydrogenase (GAPDH)*, *gamma-actin1 (ACTG1)*), which were similarly expressed in each method, cell line, and dataset (*GAPDH*: Figure 2A; *ACTG1*: Supplementary Figure S1A). In the following, we focus on the *SOX* (*=SRY-related HMG-box*) family members, especially *SOX21*. We were able to detect *SOX21* with the cDNA array; however, we observed no read counts with RNA-seq (Figure 2A, Supplementary Tables S4 and S5). Additionally, we showed these results for further cell lines to demonstrate their independence of the melanoma cell line and consistency across different array and RNA-seq datasets (Supplementary Figure S1A; Supplementary Tables S1, S2, S4 and S5). To underline the expression of *SOX21* within the melanoma cell lines and support the microarray data, we analyzed *SOX21* on the mRNA (qRT-PCR) (Figure 2B) and protein (Western blot) levels (Figure 2C) and, therefore, confirmed its expression in melanoma cell lines.

Further, we performed qRT-PCR analyses to determine whether the loss of *SOX21* in RNA-seq data was due to the library preparation, the mapping algorithm (STAR alignment software v2.5.2a, [17]), or the sequencing process [17]. We used samples produced from RNA with a standard cDNA preparation protocol and compared them with samples used for RNA-seq analysis after enrichment through PCR and ligation of the adapters (“library” or “library sample”; detailed description: Methods and Materials—Analysis of Gene Expression with Quantitative Real-Time PCR; Figure 2D). Corresponding to the RNA-seq data and in contrast to cDNA, the qRT-PCR quantification of the prepared library samples showed no results for *SOX21* for the exemplarily used cell lines, indicating that *SOX21* was lost during the sample preparation for RNA-seq. To confirm this observation, we searched for two specific *SOX21* patterns (TACATGATCCCGTGCAACTG, TTAACCTTTATGTGTAAATG) in the raw RNA-seq fastq files, allowing up to three mismatches. The *SOX21* pattern search yielded no hits with up to one mismatch. Random hits started to accumulate with two or more mismatches. A *GAPDH* pattern served as a positive control and yielded numerous hits with no mismatches. Thus, we can exclude that the detected loss of *SOX21* read counts was a mapping artifact (Figure 2E).

To investigate whether the loss of detection was observed for the whole *SOX* family, we chose additional *SOX* genes (*SOX2*, *SOX3*, *SOX4*, and *SOX11*) and compared the cDNA array data (Supplementary Table S4) with the RNA-seq read counts (Supplementary Table S5) relative to *GAPDH*. All of the *SOX* genes showed a positive signal with the cDNA microarray gene expression analysis (Figure 2F). Interestingly, the RNA-seq experiments resulted in no read counts for *SOX3* and *SOX11*, while *SOX2* and *SOX4* showed a comparable expression in RNA-seq to that observed in the cDNA arrays (Figure 2G). This was already visible within the comparison of the transcriptome data from the microarray and RNA-seq analysis (Figure 1A,B, red label) and was further confirmed in another transcriptome analysis dataset (Supplementary Figure S1B,C). We additionally quantified the mRNA levels of these *SOX* genes with qRT-PCR from cDNA in comparison with prepared library samples (Figure 2H) and observed the same aforementioned loss in our RNA-seq library samples. We were able to detect fluorescence signals for all of the investigated *SOX* genes through the microarray analysis, and we were able to quantify the cDNA expression levels; however, no RNA-seq reads were found for *SOX3* or *SOX11*, and no amplification was observed when analyzing the respective library samples with qRT-PCR. We also compared the cDNA array (Supplementary Figure S2A) and the RNA-seq data for the entire *SOX* gene family (Supplementary Figure S2B) and demonstrated that there were genes in addition to *SOX21*, *SOX3*, and *SOX11* within the *SOX* family that could not be detected with RNA-seq, but resulted in a fluorescence signal in the microarray analysis.

### 2.3. Analysis of Further Genes through Transcriptome Analysis

**Figure 3:**
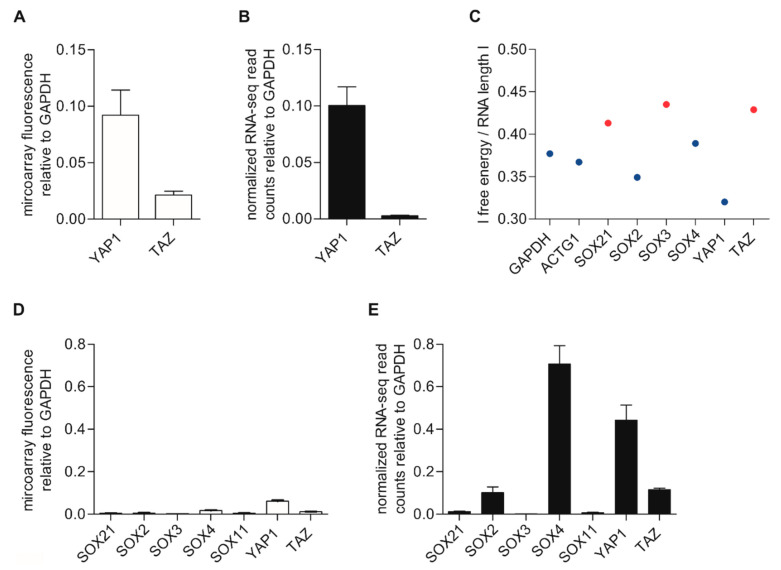
Detection of genes using transcriptome analysis. (**A**) Analysis of the microarray fluorescence signals of *YAP1* and *TAZ* (Supplementary Table S4) relative to *GAPDH* within three different cell lines. (**B**) *YAP1* and *TAZ* detection with RNA-seq (Supplementary Table S5) normalized for each cell line to *GAPDH*. (**C**) Trend for genes that are detectable by using RNA-seq (blue dots) as a function of the |free energy| divided by the RNA length. Undetectable genes via RNA-seq are illustrated as red dots. (**D**) Analysis of the microarray data from Hoek et al. (GSE4845 GPL570) for some genes of interest. (**E**) Analysis of RNA-seq datasets of mechanically fragmented RNA samples (*SOX3* mean value: 0.88 × 10^−3^) from Kunz et al. (GSE112509). *GAPDH* was used for normalization. The box plots show the mean ± SEM.

To rule out that the observed loss of gene information was mainly associated with *SOX*-transcription factors, we further analyzed *YAP1* (*yes-associated protein 1*) and *TAZ* (*transcriptional co-activator with PDZ-binding motif*) within the datasets. Therefore, we analyzed the fluorescence signals detected with the microarray analysis for *YAP1* and *TAZ* (Figure 3A) and the read counts measured with RNA-seq for both genes (Figure 3B). We were able to detect both genes with both methods (Figure 3A,B). These results were again independent of the housekeeper and were demonstrated in parallel for *ACTG1* within two different datasets of transcriptome analyses for each method (Supplementary Figure S1D,E). The microarray data indicate that genes that have generally lower detection signals (Figure 3A and Figure S1D) are potentially more difficult to detect with RNA-seq (Figure 3B and Figure S1E).

In addition, the effect of genes that are undetectable or difficult to detect with RNA-seq was not only observed for transcription factors, as shown in Figure 1A,B (black dots: n.d. RNA-seq) and the corresponding Supplementary Table S3, but also for *S100A7,* which is known in the literature to be expressed in melanoma, as well as *chemokine 5* (*CXCL5*), just to name a few [18,19,20].

### 2.4. Discussion of Possible Causes of Gene Loss

After demonstrating the loss of information by comparing the results of RNA-seq, microarrays, and qRT-PCR based on various examples and independent of the cell line and housekeeper, we aimed to understand the underlying causes for why certain genes are more difficult to detect or are not detectable with RNA-seq. First, we focused on the GC content, which could make it challenging to identify the genes investigated in this study. Price A. et al. (2017) discussed this before and observed a relationship between a varying GC content, local RNA secondary structure, and read depth [21]. However, linking the GC content to the detection of the genes of interest did not lead to conclusive results. We could not see any dependence of the GC content on the detectable or undetectable genes with RNA-seq (Supplementary Table S6). Because RNA directly reaches its folded state after synthesis, we further focused on the secondary structure of the RNA [22]. Here, we used a thermodynamic structure prediction tool to predict the minimum free-energy structures and base-pair probabilities from single RNA sequences according to the Zuker algorithm [23]. We calculated the total of the quotient out of the minimum free energy of the mRNA secondary structure and the number of nucleotides of each RNA sequence to adjust to the mRNA length (Supplementary Table S6). These additional analyses showed a lower quotient for detectable genes (Figure 3C, blue dots) and a higher quotient for undetectable genes (Figure 3C, red dots). This led to the assumption that the secondary structure could be one of the main criteria for the accessibility of the RNA for further processing steps and, consequently, for a successful RNA sequencing approach.

For the processing, library preparation, and subsequent sequencing, RNA molecules must initially be sheared into smaller pieces to be compatible with most deep sequencing technologies, such as RNA-seq [1]. There are two options for RNA shearing: chemical and mechanical [7,24]. Chemical shearing includes shearing with enzymes (RNase III), alkaline solutions, or divalent cations (Mg^++^, Zn^++^) with incubation at an elevated temperature (70 to 95 °C), while mechanical shearing comprises acoustic shearing (nebulization, sonification) [7,24,25]. It is possible that different processing methods are differently affected by the RNA secondary structure [7].

### 2.5. Analysis of Mechanically Sheared RNA-Seq Datasets

To exclude that the described observations only apply to our datasets, we analyzed already published RNA-seq data from Kunz et al. (accessible NCBI Gene Expression Omnibus (GEO), GEO Series GSE112509, melanoma tissue) and the cDNA microarray data from Hoek et al. (GEO Series accession GSE4845 GPL570, melanoma cell lines) [26,27], keeping in mind that comparing expression data obtained in different laboratories and using different biological materials can also yield discrepancies. Differently from our enzymatic shearing approach, the samples of Kunz et al. were preprocessed through mechanical shearing [26]. Due to the fact that Kunz et al. used melanoma tissue in an independent study, this comparison with our datasets only gives an approximation of the difference between the two fragmentation methods. Interestingly, the published analysis results of both the microarray (Figure 3D, Hoek et al. [27]) and RNA-seq (Figure 3E, Kunz et al. [26]) showed a different output concerning the genes of interest compared to the data analysis of our RNA-seq approach. Here, the expression of all genes of interest (*SOX2, SOX3, SOX4, SOX11, SOX21, YAP1,* and *TAZ*) could be determined with RNA-seq. We further investigated *YAP1* and *TAZ* and, in comparison with our data, were able to detect both genes through mechanical RNA sample processing (Figure 3E). We further analyzed the whole *SOX* gene family for the microarray analysis and RNA-seq of the datasets from Hoek et al. (Supplementary Figure S2C) and Kunz et al. (Supplementary Figure S2D), respectively [26,27]. By comparing the RNA-seq detection of the *SOX*-gene family with different fragmentation methods, it became clear that several genes remained measurable during mechanical processing (Supplementary Figure S2D) compared to chemical fragmentation (Supplementary Figure S2B). Therefore, it seems that chemical shearing is less effective for highly structured RNA, and there are differences within the outputs of RNA-seq with different fragmentation methods. However, this assumption remains to be verified for melanoma cell lines that are preprocessed through mechanical shearing, as the effects of other factors (such as the different biological materials used) must be kept in mind.

Consequently, as described by Griffith M. et al., the RNA-seq method is still an area under development, and changing experimental design parameters can have significant impacts on the strategy of the analysis and on the results [28]. This is also demonstrated by the possibility of using different kits from various suppliers for sample/library preparation. In summary, when using different datasets generated by different groups (e.g., provided on NCBI GEO), it is important to pay attention to the sample/library preparation and data generation, as well as to confirm the results with another method—e.g., qRT-PCR.

## 3. Materials and Methods

### 3.1. Cell Lines and Culture Conditions

Human melanoma cell lines (*501 Mel, A375, Mel Ei, Mel Ho, Mel Im, Mel Juso,* and *Mel Wei*) were described previously [29,30,31]. The human cell lines *Mel Ei, Mel Ho, Mel Im*, *Mel Juso,* and *Mel Wei* were provided by Dr. Judith Johnson (LMU, Munich, Germany). *A375* cells (*CRL-1619*) were obtained from ATCC and *501Mel* cells were provided by Dr. Ruth Halaban (Department of Dermatology, Yale University School of Medicine, New Haven, CT, USA). Cells were maintained in Dulbecco’s modified Eagle’s medium (DMEM) supplemented with penicillin (400 units∙mL^−1^), streptomycin (50 mg∙mL^−1^), and 10% fetal calf serum (FCS; Sigma Aldrich, St. Louis, MO, USA). For the melanoma cell lines *501 Mel, Mel Ho,* and *Mel Juso,* Roswell Park Memorial Institute (RPMI) 1640 medium with NaHCO_3_ was used with the same supplements. All cell lines were split at a ratio 1:5 on every 3rd day and were incubated at 37 °C in a humidified atmosphere containing 8% CO_2_. The cell lines *Mel Im* and *Mel Ju*so were analyzed with both RNA-seq and a cDNA microarray (see Section 3.4 and Section 3.5). *WM3211, WM1366,* and *WM793,* which were analyzed within a second dataset for RNA sequencing, were previously described and provided by Dr. Meenhard Herlyn (Wistar Institute, Philadelphia, PA, USA) [32]. These *Wistar* cell lines were maintained in a culture medium consisting of MCDB153 (Sigma Aldrich) with 20% Leibovitz’s L-15 (PAA Laboratories, Pasching, Austria), 2% FCS, 1.68 mM CaCl_2_ (Sigma Aldrich), and 5 µg·mL^−1^ insulin (Sigma Aldrich), and they were incubated at 37 °C in an atmosphere containing 5% CO_2_.

### 3.2. Protein Analysis (Western Blotting)

Cell lysates were prepared as described; 30 µg was loaded per lane, separated on a 12.75% sodium dodecyl sulphate polyacrylamide gel electrophoresis (SDS-page) gel, and blotted onto a polyvinylidene difluoride (PVDF) membrane (Bio-Rad) [33]. After blocking for 1 h with 5% bovine serum albumin solved in tris-buffered saline with Tween20 (BSA/TBS-T), the membrane was incubated at 4 °C overnight with one of the following antibodies: anti-SOX21 antibody (1:2000; AMAB91311; Sigma Aldrich) or anti-GAPDH (1:1000; #2118; Cell Signaling Technology, Danvers, MA, USA). After three steps of washing with TBS-T, the membrane was incubated for 1 h at room temperature with a horseradish peroxidase-coupled secondary anti-mouse or anti-rabbit antibody at 1:2000 dilution in TBS-T. After washing again, the staining was performed by using the ECL Plus Western Blotting Detection Kit (GE Healthcare Life Science Europe GmbH, Freiburg, Germany), and luminescence was measured with the Intas ECL chemocam imager.

### 3.3. Analysis of Gene Expression with Quantitative Real-Time PCR (qRT-PCR)

Isolation of total cellular RNA was performed using the E.N.Z.A. MicroElute Total RNA Kit (Omega Bio-Tek, VWR, Darmstadt, Germany) as described by the manufacturers. cDNA was generated as previously described through the use of 500 ng RNA [34]. The description “library sample” or “library” was used to designate RNA samples that were prepared for RNA-seq analysis according to the manufacturer’s instructions (Illumina, Inc., San Diego, CA, USA); they contained ligated adapters and were enriched through PCR. Therefore, 1 µL out of a 1:10 dilution of these enriched library samples was used for analysis with qRT-PCR. The qRT-PCR analysis was performed on a LightCycler^®^ 480 II system (Roche, Rotkreuz, Switzerland) as described before, and it was performed with specific sets of primers (Table 1) [35]. GAPDH was used for normalization. For each gene analysis, the length of the LightCycler product was chosen to be nearly identical to the product length of the housekeeper GAPDH. Therefore, two different GAPDH primers were required for normalization. For all qRT-PCR analyses, at least two different melanoma cell lines were used with three biological replicates.

### 3.4. Transcriptome Analysis with cDNA Microarrays

After the cellular RNA was isolated, sample processing was performed at an Affymetrix Service Provider and Core Facility, “KFB—Center of Excellence for Fluorescence Bioanalytics” (Regensburg, Germany; www.kfb-regensburg.de (accessed on 8 December 2020)). Samples were generated according to the manufacturer’s instructions for the Affymetrix GeneChip WT Plus reagent kit (Thermo Fisher Scientific, Waltham, MA, USA). The fluorescence signals were measured with an Affymetrix GeneChip Scanner 3000 7G and normalized with the RMA algorithm. In this study, two datasets were used. The first dataset contained two different cell lines (*Mel Im* and *Mel Juso*) that were analyzed in two biological replicates (Supplementary Table S1). The second dataset (Supplementary Table S4) contained three further melanoma cell lines (*501 Mel, A375,* and *Mel Ho)* in a single replicate each.

### 3.5. Transcriptome Analysis with Total RNA-Seq

RNA-seq samples and libraries were prepared as described previously [36]. Library preparation was performed with at least two biological replicates. The resulting libraries were checked for size (200–500 bp) and concentration by Tape Station 4200 (Agilent) using the High-Sensitivity DNA Kit (Agilent). Qualified RNA-seq libraries were sequenced according to the 75 bp paired-end RNA-seq approach on a HiSeq3000/4000 (Illumina, Inc.) with an average number of 20 million reads per sample. Paired-end reads were aligned to the human reference genome (hg38) and processed as described previously [37]. The resulting annotated reads normalized to library size were used for further analysis. For analysis within this manuscript, two different datasets were used. The first dataset (Supplementary Table S2) contained the same cell lines (*Mel Im* and *Mel Juso*) analyzed with a cDNA microarray. The second dataset (Supplementary Table S5) contained three additional melanoma cell lines (*WM3211, WM1366,* and *WM793*). In both datasets, the cell lines were analyzed in two biological replicates.

### 3.6. Analysis of the RNA

The free energy of the RNA secondary structure was analyzed with the RNAfold server of the ViennaRNA Web service (http://rna.tbi.univie.ac.at/ (accessed on 2 March 2021)). This tool calculates the minimum free energy of an RNA sequence based on the Zuker algorithm [23]. For calculations of the GC content of RNA molecules, the online tool endmemo (http://www.endmemo.com/bio/gc.php (accessed on 11 March 2021)) was used.

### 3.7. Statistical Analysis

The results are shown as the mean ± SEM (standard error of the mean) calculated with the GraphPad Prism software (GraphPad Software, Inc., San Diego, CA, USA). A correlation analysis was performed using R v.4.0.3 with the help of the ggplot2 and ggpubr v.0.4.0 packages by A. Kassambara (https://github.com/kassambara/ (accessed on 8 December 2020)) [38,39,40].

### 3.8. Accession Numbers

The data were deposited in the NCBI Gene Expression Omnibus under GEO:

Kunz et al. [26]; GSE112509; DESeq2_normalized_counts file: (Ensemble-ID: ENSG00000111640 = GAPDH; ENSG00000125285 = SOX21); https://www.ncbi.nlm.nih.gov/geo/query/acc.cgi?acc=GSE112509 (accessed on 11 January 2021).

Hoek et al. [27]; GSE4845-GPL570_series_matrix: (ID_REF: 212581_x_at = GAPDH; 208468_at = SOX21); https://www.ncbi.nlm.nih.gov/geo/query/acc.cgi?acc=GSE4845 (accessed on 11 January 2021).

## 4. Conclusions

This study indicates that genes with strong secondary-structured mRNA are difficult to determine in RNA sequencing after chemical shearing. Chemical shearing seems to fail in breaking up strong secondary RNA structures. However, we cannot state whether the method of chemical shearing also has advantages with regards to the detection of other genes. Therefore, we strongly suggest that, as for cDNA array analyses at the time that that method was established, all pros and cons must be defined in detail and made public; if possible, they also need to be incorporated into bioinformatical analyses. Although RNA-seq offers immense advantages over microarray analysis, there are challenges that need to be addressed. Therefore, we recommend validating the results of transcriptome analyses by using at least one additional method.

## Figures and Tables

**Table 1:** Oligonucleotide sequences for the qRT-PCR analysis.

Primer	Forward Primer 5′-3′	Reverse Primer 5′-3′	Product Size in bp	Melting Peak in °C
GAPDH	TGGGGAAGGTGAAGGTCGGA	TTGATGACAAGCTTCCCGTTC	207	83
GAPDH	GGCTCTCCAGAACATCATCCCTGC	GGGTGTCGCTGTTGAAGTCAGAGG	269	88
SOX21	GGAGAACCCCAAGATGCACA	CCGGGAAGGCGAACTTGT	202	89
SOX2	GAACCAGCGCATGGACAGTT	AGCCGTTCATGTAGGTCTGC	199	91
SOX3	GATAAGCCTACCCTTCCCGC	GTGTCCCTACGGGGTTCTTG	196	92
SOX4	CAGCAAACCAACAATGCCGA	GATCTGCGACCACACCATGA	209	93
SOX11	GAGGGCGAATTCATGGCTTG	ATTTTCCAGCGCTTGCCCAG	199	89
YAP1	CCCTCGTTTTGCCATGAACC	ACCATCCTGCTCCAGTGTTG	286	88
TAZ	TGGACCAAGTACATGAACCACC	AAATTCTGCTCCTCGGCACA	278	88

## Data Availability

External data sources used in this study are cited in the article. The extracted data are available in the Supplementary Material.

## Supplementary Materials

The following are available online at https://www.mdpi.com/article/10.3390/ijms22179349/s1.
