# Supplementary material for: Loss of Gene Information: Discrepancies between RNA Sequencing, cDNA Microarray, and qRT-PCR
Source: Int J Mol Sci. 2021 Aug 28;22(17):9349. doi: 10.3390/ijms22179349 (PMC8430810; doi:10.3390/ijms22179349)
Supplement: Supplementary file 1 [file ijms-22-09349-s001.zip › Supplementary figures table6.pdf]

International Journal of
Molecular Sciences

MDPI

Article

Supplementary Material: Loss of Gene Information: Discrepancies between RNA Sequencing, cDNA Microarray and qRT-PCR

Nicole Rachinger [1], Stefan Fischer [2], Ines Böhme [1], Lisa Linck-Paulus [1], Silke Kuphal [1], Melanie Kappelmann-Fenzl [2] and Anja K. Bosserhoff [1,*]

[1] Institute of Biochemistry, Friedrich-Alexander University Erlangen-Nürnberg (FAU), Fahrstraße 17, 91054, Erlangen, Germany
[2] Faculty of Computer Science, Deggendorf Institute of Technology, Dieter-Görlitz-Platz 1, 94469, Deggendorf, Germany
* Correspondence: anja.bosserhoff@fau.de

Supplementary Figures:

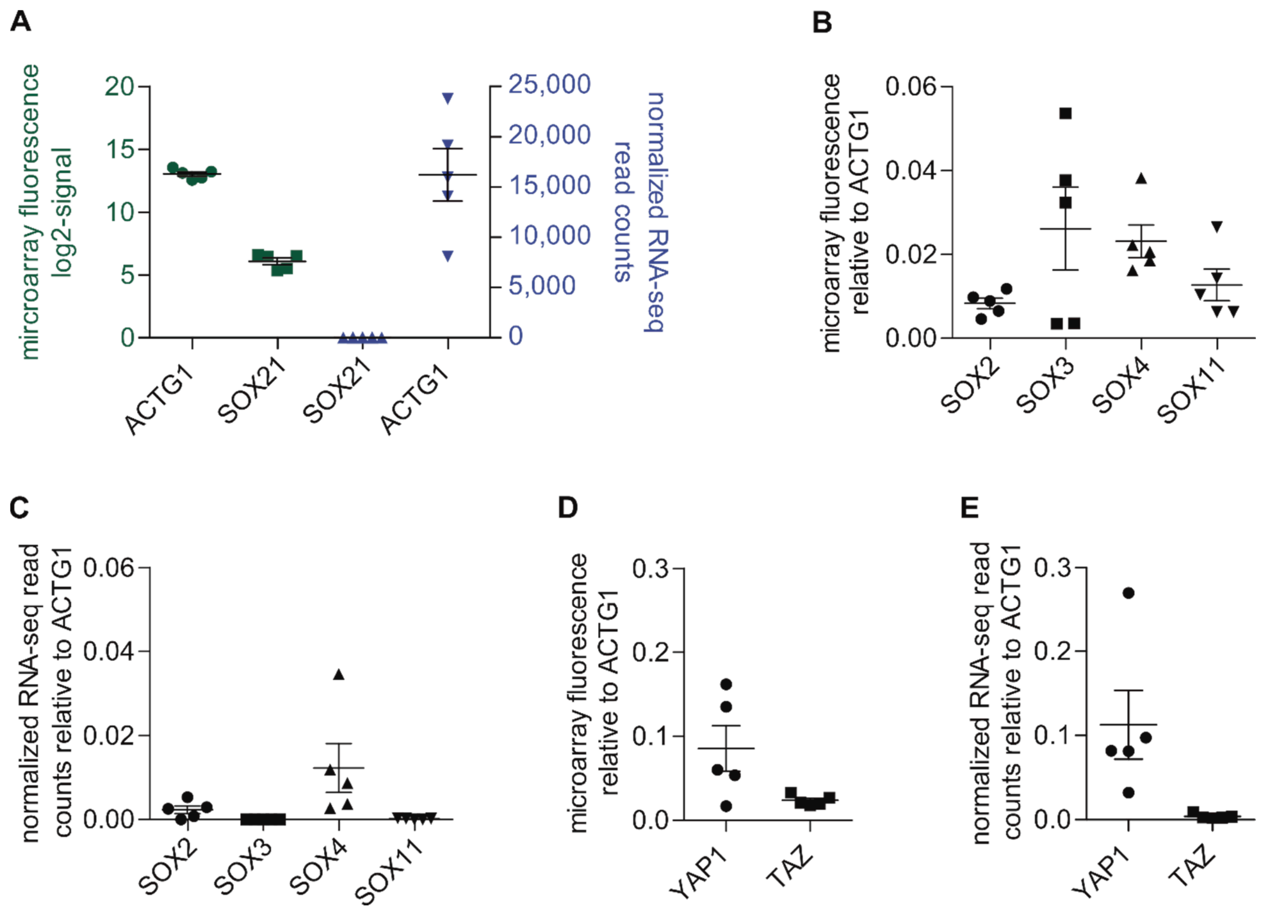

Figure S1. Comparison of the transcriptomic expression analysis by cDNA microarray and RNA-seq of different genes. A) Evaluation of the raw data from transcriptome analysis by cDNA microarray (Supplementary Table S1 and S4) and RNA-seq (Supplementary Table S2 and S5) for ACTG1 and SOX21. **B)** Analysis of the microarray detection of further SOX genes. **C)** RNA-Seq read counts depicted for further SOX gene family members. **D)** Transcriptomic profile of YAP1 and TAZ measured by microarray. **E)** Representation of RNA-seq read counts of YAP1 and TAZ. Unless otherwise declared ACTG1 was used for normalization of each method and cell line separately. Therefore datasets (cDNA microarray: Supplementary Table S1 and S4; RNA-seq: Supplementary Table S2 and S5) of five different cell lines were used. The box plots show the mean ± SEM.

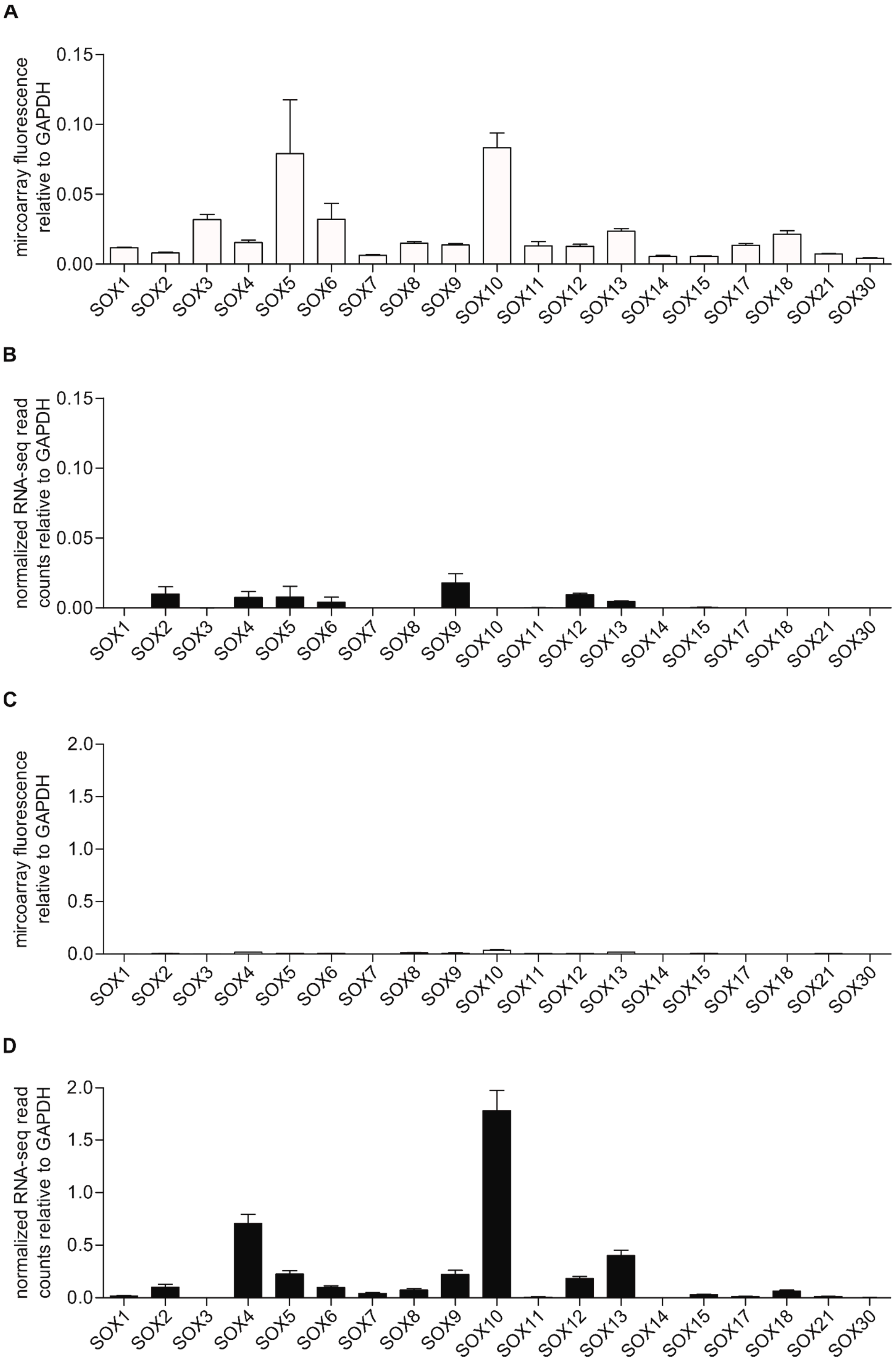
A
mircoarray fluorescence relative to GAPDH
0.15
0.10
0.05
0.00
SOX1 SOX2 SOX3 SOX4 SOX5 SOX6 SOX7 SOX8 SOX9 SOX10 SOX11 SOX12 SOX13 SOX14 SOX15 SOX17 SOX18 SOX21 SOX30
B
normalized RNA-seq read counts relative to GAPDH
0.15
0.10
0.05
0.00
SOX1 SOX2 SOX3 SOX4 SOX5 SOX6 SOX7 SOX8 SOX9 SOX10 SOX11 SOX12 SOX13 SOX14 SOX15 SOX17 SOX18 SOX21 SOX30
C
mircoarray fluorescence relative to GAPDH
2.0
1.5
1.0
0.5
0.0
SOX1 SOX2 SOX3 SOX4 SOX5 SOX6 SOX7 SOX8 SOX9 SOX10 SOX11 SOX12 SOX13 SOX14 SOX15 SOX17 SOX18 SOX21 SOX30
D
normalized RNA-seq read counts relative to GAPDH
2.0
1.5
1.0
0.5
0.0
SOX1 SOX2 SOX3 SOX4 SOX5 SOX6 SOX7 SOX8 SOX9 SOX10 SOX11 SOX12 SOX13 SOX14 SOX15 SOX17 SOX18 SOX21 SOX30

Figure S2: Comparison of the transcriptomic expression analysis for the SOX gene family by cDNA microarray and RNA-seq of different fragmentation methods. A) Detection profile analyzed by cDNA array (Supplementary Table S4) for three different melanoma cell lines. **B)** RNA-seq detection profile (Supplementary Table S5) for the SOX gene family by chemically fragmented RNA was shown. **C)** Depicts the cDNA array data from Hoek et al. (GSE4845 GPL570) for the SOX gene family profile. **D)** RNA-seq data from Kunz et al. (GSE112509) with mechanically fragmented RNA were shown. For all datasets GAPDH fluorescence signal for microarray analysis or read counts for RNA-seq analysis was used as reference. Box plots show the mean ± SEM.

Supplementary Tables:

Table S1: cDNA microarray dataset 1.

Table S2: RNA-seq dataset 1.

Table S3: Listed genes not detectable within RNA-seq dataset 1.

Table S4: cDNA microarray dataset 2.

Table S5: RNA-seq dataset 2.

Table S6: Main criteria for measurable genes by cDNA microarray and RNA-seq. Summary of expression data, GC content, free energy and length of RNA for different genes, defining a trend for the detection of genes by RNA-seq dependent on the |free energy| divided by the RNA length.

name of the gene	cDNA array [1]	normalized RNA-seq reads[1]	GC content [2] in percent	free energy [3] (RNA fold) in $kcal \cdot mol^{-1}$	length in bp (base pairs)	quotient of |free energy| to length in $kcal \cdot mol^{-1} \cdot bp^{-1}$
**GAPDH**	12,911.93	14,152.95	56.1	−484.65	1285	0.377
**ACTG1**	10,075.86	16,387.26	56.1	−748.40	2038	0.367
**SOX21**	91.39	0.72	55.6	−1208.90	2924	0.413
**SOX2**	103.78	57.36	50.7	−877.91	2512	0.349
**SOX3**	405.60	0.19	63.3	−907.24	2085	0.435
**SOX4**	164.29	97.37	52.5	−1896.38	4869	0.389
**YAP1**	1,140.21	1,383.82	43.5	−1730.04	5401	0.320
**TAZ**	268.69	39.17	60.4	−818.68	1906	0.429

[1] values out of Supplementary Table S1 and S2 rounded to two decimal places. [2] data from endmemo DNA/RNA GC content calculator. [3] calculated with Zuker algorithm by the RNAfold server of the ViennaRNA Web service.
